# Adjuvant Radiation after Primary Resection of Atypical Lipomatous Tumors of the Extremity Reduces Local Recurrence but Increases Complications: A Multicenter Evaluation

**DOI:** 10.1155/2022/2091677

**Published:** 2022-08-22

**Authors:** Joshua M. Lawrenz, Samuel R. Johnson, Kevin Zhu, Mallory McKeon, Cullen P. Moran, Jose Vega, Katherine S. Hajdu, James P. Norris, Leo Y. Luo, Eric T. Shinohara, Justin M. M. Cates, Brian P. Rubin, John D. Reith, Jennifer L. Halpern, Nathan W. Mesko, Herbert S. Schwartz, Lukas M. Nystrom, Ginger E. Holt

**Affiliations:** ^1^Department of Orthopaedic Surgery, Vanderbilt University Medical Center, Nashville 37232, TN, USA; ^2^Department of Orthopaedic Surgery, Cleveland Clinic, Cleveland 44195, OH, USA; ^3^Department of Orthopaedic Surgery, Spartanburg Regional Healthcare System, Spartanburg 29307, SC, USA; ^4^Department of Radiation Oncology, Vanderbilt University Medical Center, Nashville 37232, TN, USA; ^5^Department of Pathology, Microbiology and Immunology, Vanderbilt University Medical Center, Nashville 37232, TN, USA; ^6^Department of Pathology, Cleveland Clinic, Cleveland 44195, OH, USA

## Abstract

**Background:**

Radiation after resection of an atypical lipomatous tumor (ALT) is controversial. This study evaluates local control and complications after the first resection of ALTs of the extremity with or without adjuvant radiation.

**Methods:**

A dual institution, retrospective review of patients treated from 1995 to 2020 with first-time resection of an ALT in the extremity was performed. In total, 102 patients underwent adjuvant radiation (XRT group) and 68 patients were treated with surgery alone (no-XRT group). The median follow-up time was 4.6 years (interquartile range (IQR) 2.0–7.3 years). The median radiation dose was 60 Gy (IQR 55–66 Gy). Univariable and multivariable analyses evaluated the association of patient, tumor, and treatment variables with recurrence and complications. Kaplan–Meier analysis evaluated local recurrence-free survival (LRFS) and time to complication.

**Results:**

The overall incidence of local recurrence was 1% (1/102) in the XRT group and 24% (16/68) in the no-XRT group (*p* < 0.001). The median time-to-recurrence was 8.2 years (IQR 6.5–10.5 years). In the XRT and the no-XRT groups, 5-yr LRFS was 98% and 92% (*p*=0.21) and 10-yr LRFS was 98% and 41% (*p* < 0.001), respectively. The absence of radiation (HR = 23.63, 95% CI (3.09–180.48); *p* < 0.001) and R2 surgical resection margins (HR = 11.04, 95% CI (2.07–59.03); *p* < 0.001) incurred a 23-fold and 11-fold increased risk of local recurrence, respectively, while tumor size, depth, location, and neurovascular involvement were not found to be independent predictors of recurrence. The complication rate was 37% (38/102) in the XRT group and 10% (7/68) in the no-XRT group (*p* < 0.001). Eight patients (8/102, 8%) required surgical management for complication in the XRT group compared with two patients (2/68, 3%) in the no-XRT group (*p*=0.10). Higher radiation dose had a modest correlation with increased severity of complication (*ρ*=0.24; *p*=0.02).

**Conclusions:**

Adjuvant radiation after first-time resection of an ALT of the extremity was associated with a significantly reduced risk of local recurrence but a three-fold increase in complication rate. These data support a 10-year follow-up for these patients and inform a notable clinical trade-off if considering adjuvant radiation for this tumor with recurrent potential.

## 1. Introduction

Atypical lipomatous tumors (ALTs) by definition involve the extremity or superficial trunk. In the retroperitoneum or other deep anatomic locations, they are still considered well-differentiated liposarcomas (WDL). This distinction is important, since in contrast to WDL, ALTs are indolent, locally aggressive neoplasms that infrequently dedifferentiate and develop metastatic potential (1.5–6.5%) [1–5]. A definitive diagnosis is based on either characteristic histologic findings or confirmation of *MDM2* gene amplification [6, 7]. ALTs carry a noteworthy risk of local recurrence, ranging between 9 and 43% in the extremities and trunk [1–3, 8–11]. Due to the indolent nature of these lesions, widely accepted treatment is marginal (R1) resection similar to a lipoma [12, 13], although some centers have reported decreased rates of local recurrence with wide (R0) resection [1].

Radiation therapy has been used as an adjuvant treatment strategy to reduce the risk of local recurrence. The French Sarcoma Group reported a 10-fold reduction in the rate of local recurrence with adjuvant radiation (19.7% to 1.7% in 283 patients from 20 centers) [8]. More recently, a different group reported their recurrence outcomes in 183 patients with ALTs, but only 14% (25 patients) received radiation [9]. Radiation was not associated with a decreased time to recurrence overall but was associated with a decreased rate of second recurrence. Both prior studies included resections performed at outside facilities in their analysis and did not report complications associated with radiation [8, 9]. There is a myriad of risks associated with radiation including fibrosis, lymphedema, infection, osteonecrosis, and secondary malignancies. Due to the limited data regarding its efficacy in minimizing recurrence, the use of adjuvant radiation after the first-time resection of an ALT remains controversial.

This study was designed to answer the following questions: (1) What is the incidence of local recurrence and what factors are associated with local recurrence after first-time resection of ALTs of the extremity with or without adjuvant radiation? (2) What is the incidence of treatment complications and what factors are associated with complications after first-time resection of an ALT of the extremity with or without adjuvant radiation?

## 2. Methods

### 2.1. Patient Inclusion

A retrospective review of patients who underwent first-time surgical resection of an extremity ALT between January 1, 1995 and December 31, 2020 was performed at two sarcoma referral centers. Institutional Review Board and Data Use Agreement approval at both institutions was obtained. Patients were included based on the following criteria: age ≥18 years, extremity location, primary tumor resection occurring at study sarcoma center, diagnosis of ALT based on final resection pathology, and minimum 3 months of clinical follow-up after resection. We identified 170 patients who met these criteria; 102 received adjuvant radiation (XRT group) and 68 did not (no-XRT group).

### 2.2. Treatment and Surveillance

At both institutions, the goal of treatment of symptomatic, benign-appearing fatty tumors of the extremity is complete marginal resection, with attempts to spare all adjacent major neurovascular structures. A preoperative biopsy is rarely performed unless imaging studies suggest the possibility of dedifferentiation. At Center 1, fluorescence *in situ* hybridization (FISH) for *MDM2* gene amplification is performed on all resected extremity fatty tumors that are not definitively diagnosed as an ALT by histomorphology. At Center 2, *MDM2* FISH is performed on all resected extremity fatty tumors that are deep to fascia. Center 1 started *MDM2* FISH testing in 2012 and Center 2 started *MDM2* FISH testing in 2006. At Center 1, the use of adjuvant radiation is surgeon dependent; adjuvant radiation is rarely used for primary resections of ALT at Center 2. When used, patients are referred postoperatively after their incision has healed (approximately 3 weeks after surgery) to radiation oncology either at our institution or locally based upon the patient's preference. Surgeons at both institutions typically obtain annual surveillance magnetic resonance imaging (MRI) for 2 years after resection; clinical and/or imaging follow-up duration after 2 years is surgeon dependent. Chest imaging is not obtained routinely. Recurrent ALTs are treated with surgical resection if patients are symptomatic or if concern exists for dedifferentiation based on imaging; in asymptomatic recurrences, clinical observation is presented as an alternative. For this study, the most recent clinic visit documentation served as a final clinical follow-up.

### 2.3. Data Collection and Study Endpoints

Patient demographic and clinical data were abstracted from the electronic medical record. The following baseline clinical characteristics were recorded: age, sex, treatment center, tumor size (greatest dimension in cm), tumor location (upper or lower extremity), tumor depth (superficial or deep), tumor neurovascular involvement (yes or no), surgical resection margin status (R0/R1 or R2), *MDM2* status (positive or unavailable), and radiation dose in Gray (Gy). The treatment center refers to the location of surgery (Center 1 or 2). Tumor size, depth, and location were abstracted from the pre-resection MRI report. Tumor neurovascular involvement (NVI) was defined as at least 50% circumferential involvement of a major neurovascular structure by the tumor. R0/R1 surgical margins represented tumors that were completely resected, whereas R2 surgical margins represented tumors that were incompletely resected (or underwent intralesional surgery). We intentionally did not separately analyze R1 and R0 surgical margins, as both a complete marginal resection (R1) and a wide resection (R0) are the current standards of care for ALT, as supported in a recently published multicenter evaluation [13]. All ALTs were diagnosed by expert bone and soft tissue pathologists at both institutions. In patients who did not undergo *MDM2* testing, the diagnosis of ALT was rendered by standard histopathologic criteria, and this did not preclude inclusion in the study. No patients were included with tumors with negative *MDM2* status.

The primary endpoints of this study were local recurrence and treatment complications. Adjudication of local recurrence was based on the surgeon's follow-up clinic note and/or imaging studies performed during the surveillance period. For recurrences that underwent resection, pathology reports were reviewed for the presence of dedifferentiation. Time to local recurrence was calculated from the date of first resection to the date of documentation of local recurrence. Subsequent recurrences, if any, were also recorded. Treatment complications were defined as those that required documented medical or surgical management. Treatment complications were categorized by severity based on the level of management required: no complication, complication requiring medical management (i.e., wound care or antibiotics), and complication requiring surgical management. Complications recorded included amputation, arteritis, cellulitis, deep wound infection, dermatitis, deep venous thrombosis, fibrosis, fracture/osteonecrosis, hematoma/seroma, ileus, lymphedema, mucositis, neuropathy, ulcer, and wound breakdown. Time to surgical management for complication was calculated from the date of first resection to the date of surgical management for the complication.

### 2.4. Patient Characteristics

The study cohort consisted of 170 patients; 102 received adjuvant radiation (XRT group) and 68 did not (no-XRT group). In the XRT group, 99% (101/102) of patients were treated at Center 1 and 1% (1/102) at Center 2. In the no-XRT group, 34% (23/68) of patients were treated at Center 1 and 66% (45/68) were treated at Center 2. The mean age was 63.0 ± 12.0 years, and 51% (86/170) were female. Most tumors were located in the lower extremity (92%, 157/170) and were deep to fascia (94%, 159/170). The mean tumor size was 18.4 ± 7.8 cm. Only 38% (64/170) of tumors involved major neurovascular structures. *MDM2* amplification was confirmed in 53% of tumors (90/170) and status unavailable in 47% (80/170). R0/R1 margins were obtained in 97% of patients (165/170). Radiation dose was available for 87 of 102 patients, and the median dose was 60 Gy (IQR 55.0–66.0; range 50–70 Gy). The median follow-up time was 4.6 years (IQR 2.0–7.3 years) with a range of 3 months to 24.2 years. Twenty-five percent (42/170) of patients had <2 years of follow-up, 30% (52/170) had 2–5 years of follow-up, 35% (59/170) had 5–10 years of follow-up, and 10% (17/170) had >10 years of follow-up. A comparison of patient, tumor, and treatment characteristics of each treatment group is provided in Table 1. There was no difference between groups regarding patient age, sex, tumor location, tumor size, tumor depth, tumor NVI, *MDM2* status, and surgical resection margin status. There was an expected difference in a treatment center, as radiation was almost exclusively performed at Center 1 (*p* < 0.001). The median follow-up time was longer in the XRT group (5.0 yrs vs. 2.5 yrs) (*p*=0.02).

### 2.5. Statistical Analyses

Student's *t*-test, one-way analysis of variances (ANOVA), Wilcoxon rank-sum test, or Kruskal–Wallis test was used to compare continuous variables. Fisher's exact test was used to compare categorical variables. Post hoc Sidak tests were used to compare median radiation doses among complication severity groups. Spearman's rank correlation was used to measure the correlation between radiation dose and complication severity. Univariable and multivariable Cox proportional hazard regression were used to determine the independent association between patient, tumor, and treatment variables with local recurrence. Univariable and multivariable logistic regression were used to determine the association between patient, tumor, and treatment variables with complications requiring medical or surgical management. Initial multivariable regression models included all variables from univariable analyses. Variables were then eliminated in a stepwise manner in the order of increasing |*z*-score| until the remaining variables were significant at *α* = 0.05. Kaplan–Meier analysis was used to evaluate time to recurrence and time to surgical management for complication. The median time-to-local recurrence was determined by the logrank test. Five and ten-year local recurrence-free survival (LRFS) rates were compared using a *z*-test from calculated Kaplan–Meier survival rates. The threshold for statistical significance was *α* = 0.05. Statistical analyses were performed using Stata software (version 16.1, College Station, Texas, USA).

## 3. Results

### 3.1. Local Recurrence

**Figure 1:**
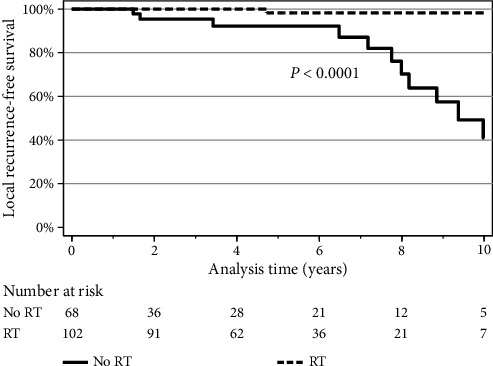
Local recurrence-free survival for XRT and no-XRT groups. The Kaplan–Meier curves show the local recurrence-free survival (LRFS) for the XRT group (dashed line) and no-XRT group (solid line). The 5-yr LRFS (95% CI) was 98% (88–99) in the XRT group and 92% (77–97) in the no-XRT group (*p*=0.21). The 10-yr LRFS was 98% (88–99) in the XRT group and 41% (17–64) in the no-XRT group (*p* < 0.001). Logrank comparison demonstrates overall difference in survival estimates (*p* < 0.0001).

The overall incidence of recurrence was 1% (1/102) in the XRT group and 24% (16/68) in the no-XRT group (*p* < 0.001) (Table 2). In the XRT and the no-XRT groups, the 5-yr LRFS was 98% and 92% (*p*=0.21) and the 10-yr LRFS was 98% and 41%, respectively (*p* < 0.001) (Figure 1). The median time-to-recurrence was 8.2 years (IQR 6.5–10.5 years) overall, 8.5 years (IQR 6.8–10.9 years) in the no-XRT group, and 4.7 years for the single patient in the XRT group. There were four recurrences (4/17, 24%) within 5 years after resection, eight recurrences (8/17, 47%) between 5 and 10 years after resection, and five recurrences (5/17, 29%) greater than 10 years after resection. No recurrent tumor showed evidence of dedifferentiation.

Univariable analysis showed clinically relevant variables including tumor size, tumor depth, tumor location, and tumor NVI to have no association with recurrence (Table 3). Multivariable analysis showed the absence of radiation (HR = 23.63, 95% CI (3.09–180.48); *p* < 0.001) and R2 surgical resection margins (HR = 11.04, 95% CI (2.07–59.03); *p* < 0.001) to incur a 23-fold and 11-fold increased hazard of local recurrence, respectively. Five patients had R2 surgical resection margins; all five tumors were deep and four of five tumors had NVI. Two of four (50%) patients in the no-XRT group with R2 surgical resection margins recurred, whereas the single patient in the XRT group with R2 surgical resection margins did not recur. In the no-XRT group, 15 of 16 (94%) patients had re-resection for recurrence, and four patients had >1 recurrence (three patients with two recurrences and one patient with four recurrences). In the XRT group, the single patient who recurred underwent re-resection and did not have another recurrence at the last follow-up.

### 3.2. Treatment Complications

**Figure 2:**
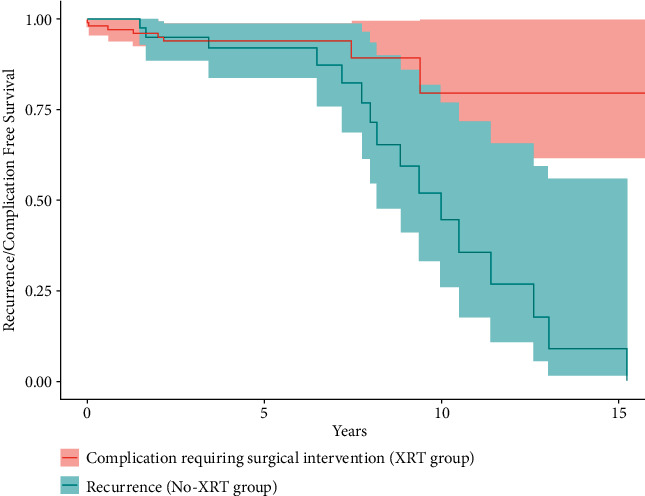
Time to recurrence or complication. Time to recurrence in 16 patients in the no-XRT group in blue (median 8.5 years (IQR 6.8–10.9)) is compared to time to surgical intervention for complication in eight patients in the XRT group in red (median 20.0 months (IQR 5.5–42.0)). Logrank comparison demonstrates overall difference in survival estimates *p*=0.01.

The overall incidence of treatment complication was 37% (38/102) in the XRT group and 10% (7/68) in the no-XRT group (*p* < 0.001) (Table 4). Eight patients (8/102, 8%) in the XRT group required surgical management for complication (three deep infections, three radiation-associated fractures, one hematoma, and one anal fissure) compared with two patients (2/68, 3%) in the no-XRT group (*p*=0.10). The median time-to-surgical management for complication was 0.8 months in the no-XRT group and 20.0 months in the XRT group (Figure 2). Lymphedema (28%, 28/102) and fibrosis (16%, 16/102) were more common in the XRT group (*p* < 0.001). There were no postradiation sarcomas.

**Figure 3:**
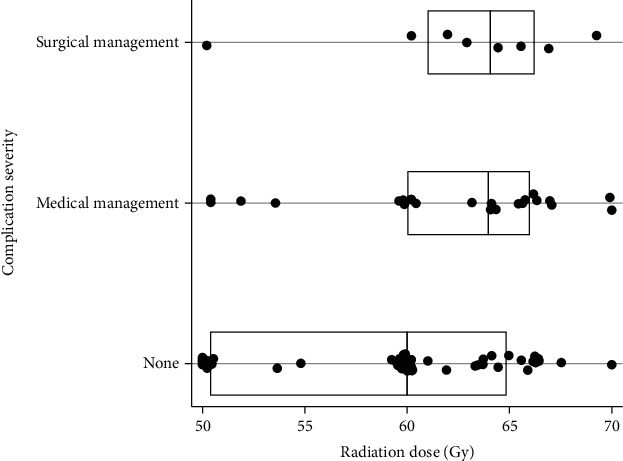
Complication severity by radiation dose. The box plot shows the median radiation dose received for patients based upon their complication severity (no complication, medical management required, and surgical management required). Eighty-seven of 102 patients had radiation dose data. Each dot represents a single patient's radiation dose. The vertical line within each box represents the median dose for that severity group, and the left and right ends of the box represent the interquartile range (IQR). The median radiation dose received for all patients was 60 Gy (55–66). The median doses for the none, medical management, and surgical management complication groups were 60 Gy (50.4–64.9), 64 Gy (60–66), and 64.1 (61–66.3), respectively. No differences in dose were found between the three groups (*p*=0.11). Spearman's rank correlation between radiation dose and complication severity was *ρ*=0.24(*p*=0.02), indicating a modest positive correlation.

Univariable analysis showed tumor size (*p*=0.009), treatment center (*p* < 0.001), radiation status (*p* < 0.001), and radiation dose (*p*=0.002) to be associated with a complication requiring medical or surgical management (Table 5). Multivariable analysis showed radiation (HR = 4.77, 95% CI (1.96–11.62)) and increased tumor size (HR = 1.31, 95% CI (1.03–1.66)) to incur a 5-fold and 1.3-fold (for every 5 cm increase in tumor size) increased odds of complication requiring medical or surgical management, respectively. A modest positive correlation was found between increasing radiation dose and complication severity (*ρ*=0.24(*p*=0.02)) (Figure 3). In patients treated with a total dose equaling 50 Gy, 19% (3/16) had a complication requiring medical or surgical management, whereas 39% (28/71) of patients receiving >50 Gy had a complication requiring medical or surgical management (*p*=0.15).

## 4. Discussion

This study sought to assess the impact of adjuvant radiation on local recurrence and treatment complications after first-time resection of ALTs of the extremity at two large sarcoma centers. Although treatment for ALTs does not typically include radiation after first-time resection, the tendency for local recurrence prompts its consideration. Prior studies regarding this issue are limited by small patient cohorts receiving radiation, inclusion of patients with resection at non-sarcoma centers, and lack of reporting of associated complications. In this study, we found that patients receiving adjuvant radiation after the first tumor resection had a significantly improved 10-yr LRFS (98% vs. 41%, *p* < 0.001). However, this treatment group suffered a three-fold increase in complication rate requiring medical or surgical management (37% vs. 10%, *p* < 0.001).

A key finding of this study of the largest cohort of patients radiated after the first resection of ALT is the improved local control in patients receiving adjuvant radiation. Recurrence in non-radiated patients was common (24%) and consistent with prior reported recurrence rates (9–43%) [1, 2, 8–10]. In addition, recurrences also occurred rather late during follow-up (median 8.2 years after resection). This parallels prior reports demonstrating delayed recurrence times (5 years [10], 7.2 years [1], and 18 years [9]) after surgery. The longest time to recurrence in our study was 15 years after surgery, while some have reported as late as 40 years after surgery [10]. These data suggest we should counsel patients that the risk of recurrence is most common between 5 and 10 years after resection and even continues beyond 10 years. These data failed to demonstrate associations between tumor size, anatomic location, depth, *MDM2* status, or neurovascular involvement with increased risk of recurrence. Outside of radiation status, R2 surgical resection margin status was the only variable associated with increased recurrence risk. Kalimuthu et al. similarly emphasized the importance of marginal or wide resection when possible, reporting worse local control with intralesional resection, which parallels the trend seen in our data [10]. A recent multicenter study further demonstrated no difference in recurrence-free survival when comparing R1 resection status to R0 resection status, emphasizing that microscopically positive margins were not associated with increased risk of recurrence [13]. Furthermore, dedifferentiation did not occur in any tumor recurrence in our study, consistent with prior studies reporting a low dedifferentiation rate of 3-4% [1, 2]. Lastly, though not specifically surveyed with routine chest imaging in this study, there were no documented lung metastases at final follow-up based on a review of medical records. This finding supports the recent findings of a study by Lazarides et al. recommending against routine chest imaging in patients with an ALT [3].

The second key finding is the treatment complication profile in patients who received adjuvant radiation. Complication characteristics of adjuvant radiation to the extremity such as lymphedema and fibrosis were quite frequent (16–28% of patients) and not unexpected. Patients receiving radiation had a more than three-fold increased incidence (37% vs. 10%) of a complication warranting some form of medical or surgical management. More noteworthy were the three deep wound infections, three femur fractures from radiation osteonecrosis, one hematoma, and one anal fissure that all required surgical management a median of 20 months after resection. Of these eight patients who received radiation and required surgical management for complication, seven received radiation doses greater than 60 Gy and had a tumor size greater than 15 cm. Given this finding and the trend in this study toward a decreased risk of complication in patients receiving 50 Gy compared to >50 Gy (19% vs. 38%, *p*=0.15), further study of the use of 50 Gy in patients who may benefit from adjuvant radiation could be considered. Outside of increasing tumor size, no other variables were associated with increased risk of postoperative complication. However, larger/deeper tumors theoretically require higher treatment volumes, which was not an association accounted for in our investigation. No patient was identified to have a radiation-associated sarcoma, although median follow-up may not have been long enough to encompass the highest risk timeframe for the development of these malignancies [14].

The findings of this study inform the clinical trade-off of radiation use after the first resection of ALTs of the extremity. While local control was improved considerably, complications for some were significant. These data raise the question as to the relative importance of preventing recurrence versus limiting complications in the setting of a benign, albeit potentially locally, aggressive tumor. It is always preferable to avoid additional therapies, particularly ones with adverse effects, if not compromising overall treatment efficacy. Some clinicians may consider radiation used for a nonmalignant tumor to be putting patients at unnecessary risk for complication. Others may argue a 57% difference (98% vs. 41%, *p* < 0.001) in 10-yr LRFS is unacceptable and the potential improvement in local control is worth the morbidity associated with radiation. This is especially true when one considers that a repeat resection of a recurrence may require more muscle to be sacrificed in the case of a deep ALT, possibly resulting in a greater postoperative functional deficit. Examples of other nonmalignant, but locally aggressive, tumors for which radiation has shown local control efficacy include desmoid tumor [15] and diffuse-type tenosynovial giant cell tumor [16]. Characterizing this clinical trade-off further, Figure 2 compares the time to surgical management for complication in radiated patients (20 months) to the time to recurrence in non-radiated patients (8.5 years), recognizing that time to event differences may also contribute to risk tolerance when deciding for or against radiation. Therefore, although these data do not conclude for or against the use of adjuvant radiation, they can be used to inform clinicians regarding the risks and benefits and help with discussing treatment options with patients. These data also bring to interest the use of alternative reduced dosing strategies for adjuvant radiation instead of the typical 60–70 Gy of conventionally fractionated radiation used for extremity sarcoma (and used in this study). Future studies could assess the effectiveness of 50 Gy of conventionally fractionated radiation given in the adjuvant setting or shorter courses of hypofractionated radiation with a similar biologically effective dose, to find the most acceptable trade-off of local control rate and complication profile.

A key limitation in this study is the follow-up bias inherent in censoring patients undergoing surveillance of nonmalignant tumors. Once treated and recovering without issue, many patients with benign tumors may only return for follow-up if a problem arises, regardless if follow-up is recommended or scheduled. The median follow-up time was 4.6 years (IQR 2.0–7.3 years), indicating that many patients were last seen in our practice before the most likely time of recurrence (5 to 10 years after surgery). Nonetheless, the overall recurrence rate in this study was similar to reported rates in prior studies, and time-dependent LRFS estimations and Cox hazard modeling were used for this reason. A second limitation pertains to the selection of patients in the study cohort. Eighty patients (47%) did not have molecular confirmation with *MDM2* FISH, as this test was unavailable at the two study centers until 2006 and 2012. Though *MDM2* was not performed on all patients, inclusion in this study required unequivocal diagnostic histopathology read by musculoskeletal trained soft tissue pathologists at both centers. It is possible that some of these patients without *MDM2* testing had lipomas or lipoma variants and not *bona fide* ALT, though prior studies have demonstrated that ALTs can be diagnosed by histologic criteria alone [5, 17]. In this study, the proportion of patients without *MDM2* results was not significantly different in the XRT and no-XRT groups, thereby theoretically lessening the impact of potential selection bias, and *MDM2* status was not found to be an independent predictor of recurrence in our multivariable analysis. Other recent studies of ALT have shown histologic criteria without *MDM2* testing to be an acceptable definition for patient inclusion, as Lazarides et al. also demonstrated no difference in recurrence rate between *MDM2*-positive tumors and those in which *MDM2* FISH was not performed [3]. A final limitation of this study is the inability to account for the variability of radiation delivery. While some patients were treated at the same center where the tumors were resected, others were referred to local radiation oncology teams; thus, the rationale for choosing the final total dose delivery to each patient cannot be further addressed. In addition, total treatment volume (dictated by both size of tumor/surgical field and added margins in the radiation planning) and use of more contemporary techniques (such as intensity-modulated radiation therapy, IMRT) were not accounted for.

## 5. Conclusion

Adjuvant radiation after primary resection of ALTs was associated with a significant reduction in local recurrence but came at the expense of a three-fold increased risk of treatment complication requiring medical or surgical management. The median time-to-recurrence was around 8 years after resection, indicating that longer clinical follow-up to 10 years may be warranted for ALT. No recurrent tumor showed evidence of dedifferentiation in our study. The authors do not recommend basing treatment decisions solely on these results; these decisions are best made after physician-patient discussions on a case-by-case basis and ideally within a multidisciplinary tumor board. However, these data help inform patients about the relative risks and benefits of adjuvant radiation when considering its use after resection of ALT.

## Figures and Tables

**Table 1:** Patient, tumor, and treatment characteristics.

Variable	XRT *n* = 102	No-XRT *n* = 68	*p*-value
Age, years, mean ± SD	62.1 ± 11.3	64.3 ± 12.9	0.25

Sex			0.16
Male	55 (54)	29 (43)	
Female	47 (46)	39 (57)	

Tumor location			0.38
Upper extremity	6 (6)	7 (10)	
Lower extremity	96 (94)	61 (90)	

Tumor size, cm, mean ± SD	19.2 ± 7.8	17.1 ± 7.8	0.09

*MDM2* status			0.06
Positive	48 (47)	42 (62)	
N/A	54 (53)	26 (38)	

Tumor depth			0.35
Deep	97 (95)	62 (91)	
Superficial	5 (5)	6 (9)	

Tumor NVI			0.20
Yes	34 (33)	30 (44)	
No	68 (67)	38 (56)	

Surgical resection margins			0.08
R0/R1	101 (99)	64 (94)	
R2	1 (1)	4 (6)	

Treatment center			**<0.001**
Center 1	101 (99)	23 (34)	
Center 2	1 (1)	45 (66)	

Radiation dose, Gy^*∗*^	60.0 (55.0–66.0)	—	—

Follow-up, years^*∗*^	5.0 (2.7–7.0)	2.5 (1.1–7.4)	**0.02**

Values presented as *n* (%), unless otherwise specified. XRT, radiation group; No-XRT, no radiation group; SD, standard deviation; N/A, not available; NVI, neurovascular involvement; Gy, gray; #, median (interquartile range).

**Table 2:** Recurrence outcomes.

	XRT *n* = 102	No-XRT *n* = 68	*p*-value
Recurrence	1 (1)	16 (24)	**<0.001**
Time to recurrence, years^*∗*^	4.7^#^	8.5 (6.8–10.9)	—
5-year LR-free survival (95% CI)	98% (88–99)	92% (77–97)	0.21
10-year LR-free survival (95% CI)	98% (88–99)	41% (17–64)	**<0.001**

Values presented as *n* (%), unless otherwise specified. XRT, radiation group; No-XRT, no radiation group; LR, local recurrence; CI, confidence interval; ^*∗*^, median (IQR); ^#^, single patient.

**Table 3:** Univariate and multivariate Cox regression analyses of patient, tumor, and treatment characteristics with recurrence.

	Univariate analysis		Multivariate analysis	
	HR (95% CI)	*p*-value	HR (95% CI)	*p*-value
Age (10-year intervals)	1.09 (0.80–1.48)	0.58	—	—

Sex				
Male	1.20 (0.45–3.18)	0.72	—	—
Female	Ref			

Tumor location				
Upper extremity	5.71 (1.46–22.34)	0.03	—	—
Lower extremity	Ref			

Tumor size (5-cm intervals)	0.85 (0.57–1.28)	0.43	—	—

*MDM2* status				
Positive	Ref			
Unavailable	0.28 (0.10–0.81)	0.01	—	—

Tumor depth				
Deep	Ref			
Superficial	0	0.35	—	—

Tumor NVI				
Yes	2.35 (0.89–6.25)	0.08	—	—
No	Ref			

Surgical resection margins				
R0/R1	Ref			
R2	9.88 (2.02–48.38)	0.02	11.04 (2.07–59.03)	**<0.001**

Treatment center				
Center 1	Ref			
Center 2	4.56 (1.64–12.65)	0.003	—	—

Radiation				
Yes	Ref			
No	23.22 (3.05– 176.94)	<0.001	23.63 (3.09–180.48)	**<0.001**

HR, hazard ratio; CI, confidence interval; ref, reference group; NVI, neurovascular involvement.

**Table 4:** Complication outcomes.

	XRT *n* = 102	No-XRT *n* = 68	*p*-value
Patients with complication by severity			**<0.001**
None	64 (63)	61 (90)	**<0.001**
Medical management required	30 (29)	5 (7)	**<0.001**
Surgical management required	8 (8)	2 (3)	0.10
Time to surgical management, months^*∗*^	20.0 (5.5–42.0)	0.8 (0.7–0.8)	—
Complication type			
Amputation	1 (1)	0 (0)	1
Arteritis	2 (2)	0 (0)	0.52
Cellulitis	6 (6)	1 (1)	0.24
Deep wound infection	5 (5)	2 (3)	0.7
Dermatitis	6 (6)	0 (0)	0.08
Deep venous thrombosis	2 (2)	0 (0)	0.52
Fibrosis	16 (16)	0 (0)	**<0.001**
Fracture/osteonecrosis	5 (5)	0 (0)	0.16
Hematoma/seroma	10 (10)	2 (3)	0.13
Ileus	0 (0)	1 (1)	0.40
Lymphedema	28 (28)	0 (0)	**<0.001**
Mucositis	1 (1)	0 (0)	1
Neuropathy	4 (4)	2 (3)	1
Ulcer	1 (1)	0 (0)	1
Wound breakdown	4 (4)	3 (4)	1
Total complications	91	11	—

Values presented as *n* (%), unless otherwise specified. XRT, radiation group; No-XRT, no radiation group; ^*∗*^, median (interquartile range).

**Table 5:** Univariate and multivariate logistic regression analyses of patient characteristics with complication requiring medical or surgical management.

	Univariate analysis		Multivariate analysis	
	OR (95% CI)	*p*-value	OR (95% CI)	*p*-value
Age (10-year intervals)	1.21 (0.90–1.61)	0.20	—	—

Sex				
Male	1.58 (0.79–3.15)	0.19	—	—
Female	Ref			

Tumor location				
Upper extremity	0.21 (0.03–1.70)	0.07	—	—
Lower extremity	Ref			

Tumor size (5-cm intervals)	1.35 (1.07–1.69)	0.009	1.31 (1.03–1.66)	**0.03**

*MDM2* status				
Positive	Ref			
Unavailable	1.25 (0.63–2.47)	0.53	—	—

Tumor depth				
Deep	Ref			
Superficial	0.60 (0.12–2.89)	0.50	—	—

Tumor NVI				
Yes	1.14 (0.57–2.30)	0.70	—	—
No	Ref			

Surgical resection margins				
R0/R1	Ref			
R2	1	—	—	—

Treatment center				
Center 1	Ref			
Center 2	0.14 (0.04–0.47)	<0.001	—	—

Radiation				
Yes	5.17 (2.15–12.46)	<0.001	4.77 (1.96–11.62)	**<0.001**
No	Ref			

Radiation dose (10 Gy intervals)	1.20 (1.07–1.35)	0.002	—	—

OR, odds ratio; CI, confidence interval; ref, reference group; NVI, neurovascular involvement; Gy, gray.

## Data Availability

The data that support the findings of this study are available from the corresponding author upon request.
